# Supplementary material for: Rapidly evolving orphan immunity genes protect human gut bacteria from intoxication by the type VI secretion system
Source: bioRxiv. 2025 May 4:2025.05.03.651265. Preprint. [Version 2] doi: 10.1101/2025.05.03.651265 (PMC12247911; doi:10.1101/2025.05.03.651265)

Supplemental Figure 1

A

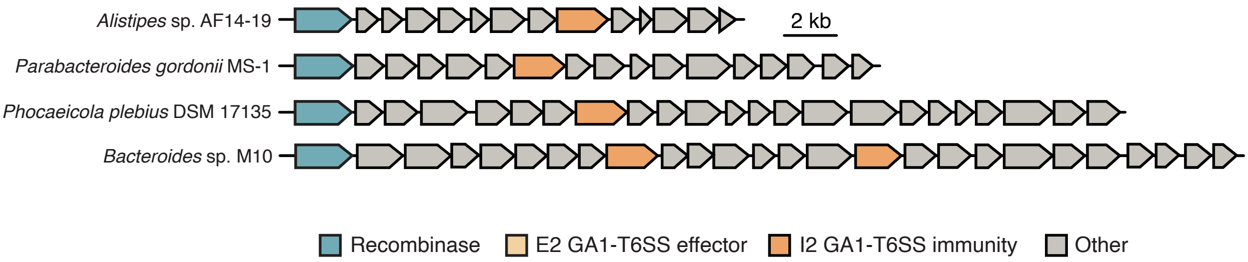

Supplemental Figure 2

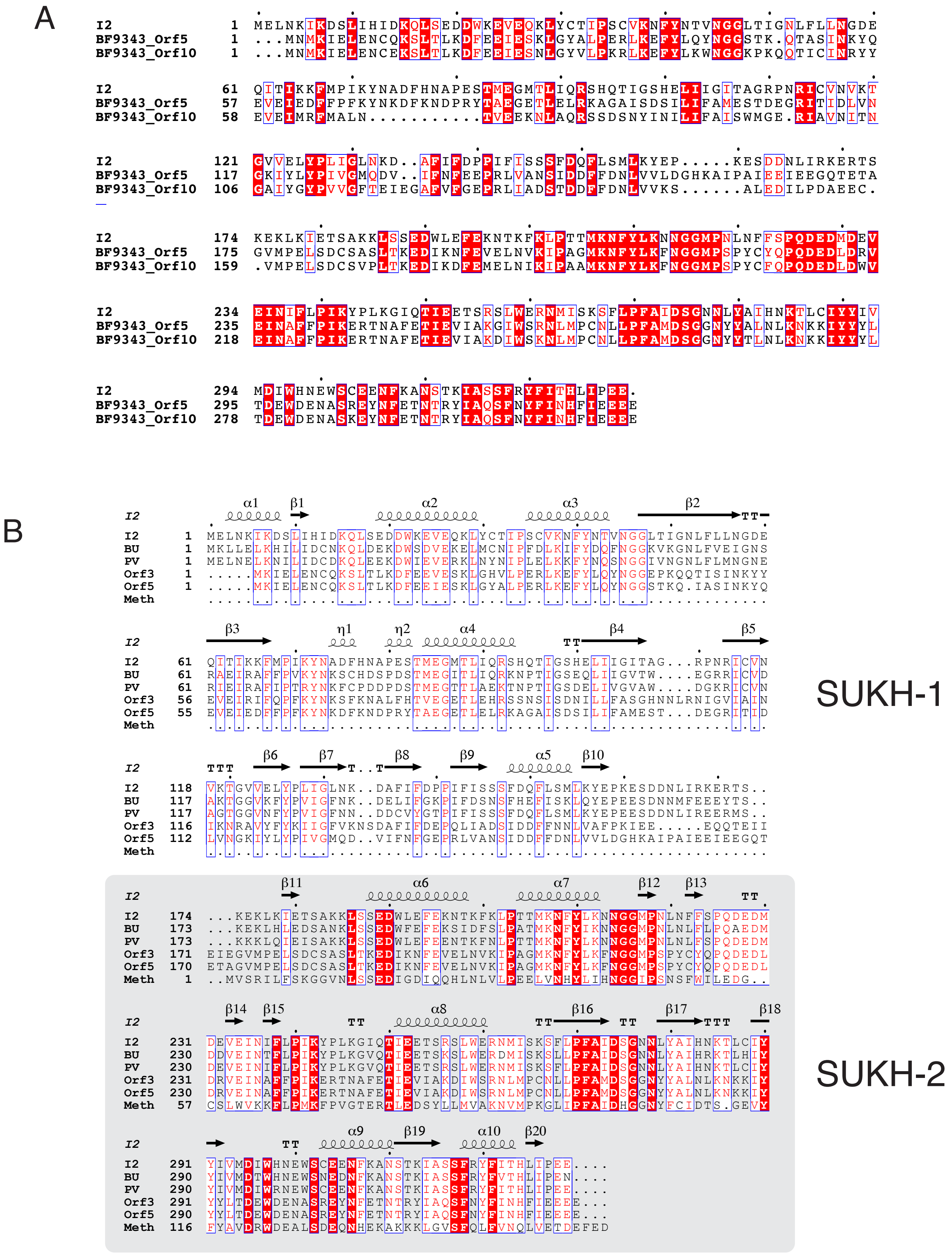

Supplemental Figure 3

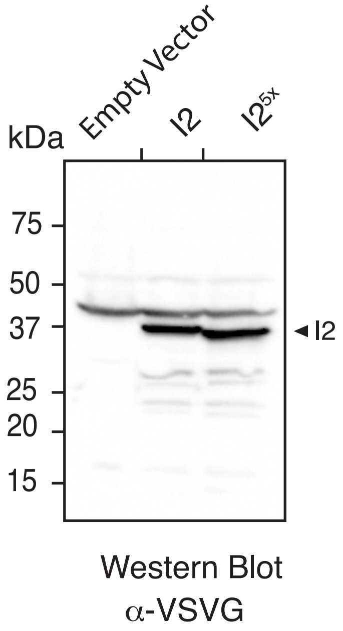

Supplemental Figure 4

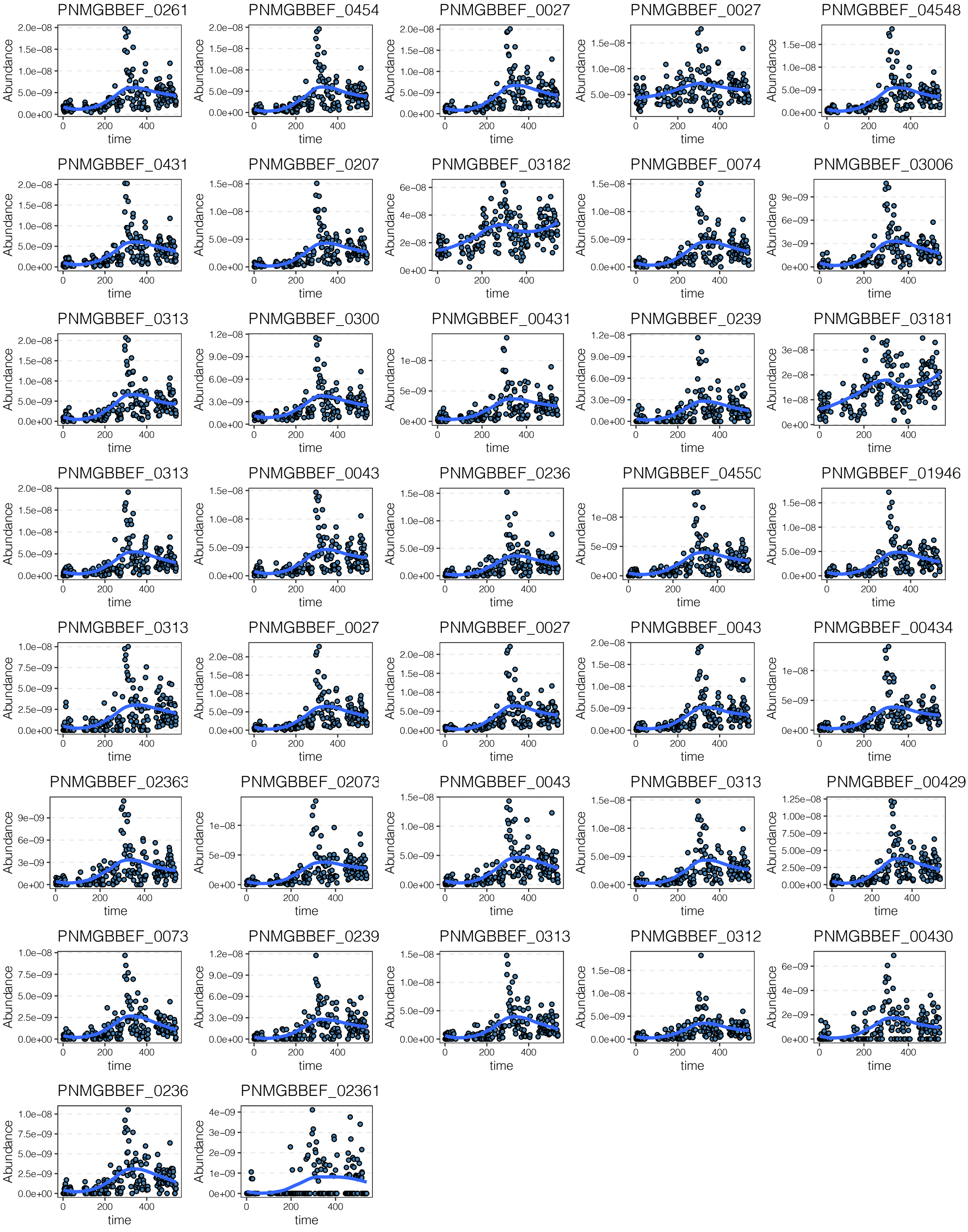

Supplement: Supplement 1 — Supplemental Figure 1. Example rAID systems. Selected rAID systems harboring orphan I2 genes from reference genomes of Bacteroidales. Supplemental Figure 2. Alignment of GA1_I2 amino acid sequences. A) Amino acid sequence alignment of cognate GA1_I2 and orphan I2 genes from the rAID system of B. fragilis NCTC 9343. B) Amino acid sequence alignment of cognate GA1_I2 sequences from B. uniformis and P. vulgatus (BU and PV), rAID orphan immunity from S01-am (orf3 and orf5), and the single domain SUKH protein from Methylomonas sp. LW13 (Meth). Supplemental Figure 3. Western blot assaying for the expression of I25x. Western blot demonstrating equivalent levels of protein in lysates from E. coli cells that express WT and mutated (I25x) proteins using anti-VSVG. Supplemental Figure 4. Abundance plots of marker genes detected in longitudinal samples from S01-am. [file media-1.pdf]
